# Mass transfer vectors for nitric oxide removal through biological treatments

**DOI:** 10.1007/s11356-023-30009-6

**Published:** 2023-10-02

**Authors:** David Fernando Cubides Páez, Xavier Guimerà Villalba, Nerea Abasolo Zabalo, Helena Torrell Galceran, Irene Jubany Güell, Xavier Gamisans Noguera

**Affiliations:** 1 Eurecat, Centre Tecnològic de Catalunya, Sustainability Area, 08243 Manresa, Spain; 2 Department of Mining, Industrial and ICT Engineering (EMIT), Biological Treatment of Gaseous Pollutants and Odours Group (BIOGAP), Manresa School of Engineering (EPSEM), Universitat Politècnica de Catalunya (UPC), Av. Bases de Manresa 61-73, 08242 Manresa, Spain; 3 Eurecat, Centre Tecnològic de Catalunya, Centre for Omic Sciences (COS), Joint Unit Universitat Rovira i Virgili-EURECAT, Unique Scientific and Technical Infrastructures (ICTS), 43204 Reus, Spain

**Keywords:** Nitric oxide, Non-aqueous phase liquids, Chemical absorption–biological reduction, Mass transfer vectors, Biological treatment, Air pollution

## Abstract

The reduction of nitric oxide (NO) emissions to atmosphere has been recently addressed using biological technologies. However, NO removal through bioprocesses is quite challenging due to the low solubility of NO in water. Therefore, the abatement of NO emissions might be improved by adding a chelating agent or a mass transfer vector (MTV) to increase the solubility of this pollutant into the aqueous phase where the bioprocess takes place. This research seeks to assess the performance of different non-aqueous phase liquids (NAPs): n-hexadecane (HEX), diethyl sebacate (DSE), 1,1,1,3,5,5,5-heptamethyl-trisiloxane (HTX), 2,2,4,4,6,8,8-heptamethylnonane (HNO), and high temperature silicone oil (SO) in chemical absorption–biological reduction (CABR) integrated systems. The results showed that HNO and HTX had the maximum gas-liquid mass transfer capacity, being 0.32 mol NO/kmol NAP and 0.29 mol NO/kmol NAP, respectively. When an aqueous phase was added to the system, the mass transfer gas–liquid of NO was increased, with HTX reaching a removal efficiency of 82 ± 3% NO with water, and 88 ± 6% with a phosphate buffer solution. All NAPs were tested for short-term toxicity assessment and resulted neither toxic nor inhibitory for the biological activity (denitrification). DSE was found to be biodegradable, which could limit its applicability in biological processes for gas treatment. Finally, in the CABR system tests, it was shown that NO elimination improved in a short time (30 min) when the three mass transfer vectors (HEX, HTX, HNO) were added to enriched denitrifying bacteria.

**Supplementary Information:**

The online version contains supplementary material available at 10.1007/s11356-023-30009-6.

## Introduction

In recent years, the majority of NO_x_ emissions are attributed to anthropogenic activities. These emissions primarily originate from the combustion of fossil fuels in industrial processes, transportation systems, and power generation facilities. In contrast, biogenic sources, including soil emissions and microbial processes, are estimated to account for less than 10% of the total NO_x_ emissions (EPA 1999). In industrial combustion processes, it was documented that approximately 95% of total NO_x_ emissions consist of NO and 5% of NO_2_, even when the O_2_ concentration ranges from 5 to 10% (Baukal 2005; Wang et al. 2013). This high NO percentage can be attributed to the limited rate of the oxidation reaction that converts NO to NO_2_ (Atkinson et al. 2004). Despite the abundance of O_2_, the oxidation kinetics are determined by factors such as temperature, pressure, and the absence of catalytic agents (Zhu and Xu 2022). Consequently, the effluent gas streams from such combustion processes are predominantly enriched in NO instead of NO_2._

NO_x_ can directly and indirectly contribute to both the eutrophication and acidification of ecosystems. NO serves as a precursor to NO_2_, which subsequently forms nitric acid (HNO_3_) (Skalska et al. 2010). These acidic compounds can be deposited into terrestrial and aquatic ecosystems via both wet and dry deposition mechanisms, thereby increasing the pool of bioavailable nitrogen in these systems. Additionally, the inhalation of NO_x_ can result in adverse health effects (Canfield et al. 2010). Currently, the main physicochemical technologies being used for controlling NO_x_ emissions from combustion gases are selective non catalytic reduction (SNCR), and selective catalytic reduction (SCR)(Wei et al. 2019). However, these techniques have two main drawbacks: their high cost, as they use expensive reagents or additional fuel as energy sources, and their high environmental impact, due to the generation of hazardous waste, such as exhausted catalysts or residual ammonia (Commission et al. 2019).

Therefore, the development of low-cost and sustainable alternatives for the treatment of NO emissions is required. In this sense, biological treatment is currently proposed as an alternative to physicochemical technologies. However, biological treatments for NO reduction have low efficiency in steady states due to the low solubility of NO (0.00618 g NO·100 g^–1^ H_2_O) (Niu and Leung 2010). One of the most widely studied (at lab-scale) bio-based alternative is the chemical absorption and biological reduction (CABR) process, which consists of a NO transfer process from the gas phase to the liquid phase followed by a biological conversion to N_2_. The most studied process for the transfer of the NO to the liquid phase is the complexation with a chelating agent, specifically Fe(II)EDTA^2−^. However, it has been reported that the regeneration of Fe(II)EDTA^2−^ through denitrifying bacteria and reducing iron can be inhibited by changes in pH, oxygen, temperature, sulfur compounds, and Fe(III)EDTA^−^, (Chandrashekhar et al. 2015) which can change the kinetics and thermodynamics of the complexation reaction (Gambardella et al. 2005).

In addition to Fe(II)EDTA^2−^, other complexed amino carboxylates of the iron family has been investigated. For examples, 2-[bis(carboxymethyl)amino] acetic acid (NTA) and 2-hydroxypropane-1,2,3-tricarboxylate (citrate) were identified as a more economic and environmentally friendly options than EDTA complexes (Liu et al. 2011). However, these alternatives had lower complexation capacity and faster degradation rates (Xu and Chang 2007; Chandrashekhar and Pandey 2017). In recent years, cobalt complexes with amino acid ligands such as cobalt (II) histidine (CoHis) have been studied because they can complex NO and O_2_ at neutral pH. Nevertheless, Co (II) is a heavy metal that can be toxic to microorganisms. Sun et al. (2019) found a maximum non-toxic concentration of 20 mM of CoHis for denitrifying bacteria and, thus, suitable to CABR system. Other options tested for NO absorption are ionic liquids (ILs) such as trihexyl (tetradecyl) phosphonium phenyl sulphonate [P_66614_] [PhSO3] (Cao et al. 2020) and deep eutectic solvents (DESs) such as ethylene glycol tetraethylenepentamine chloride (EG-[TEPA]Cl (3:1)) (Sun et al. 2020). Despite having good absorption capacity, their application in bio-based technologies is unfeasible in the short term because their biodegradability, toxicity, and stability are still unknown. Furthermore, the applicability of ILs and DESs in biological processes is still unclear because in the presence of water, the absorption capacity may be reduced (Wazeer et al. 2021).

In the review by Cubides et al. (2023), a detailed list of the mass transfer vector (MTV) options to improve NO absorption, and the degree of development of each solution, is presented. The authors concluded that there is still not a clear option that has been proven to be industrially scalable. A promising option is the use of water immiscible liquids with high affinity for poorly water-soluble compounds such as organic solvents, called non-aqueous phase liquid (NAPs) (Quijano et al. 2009).

Different NAPs have been studied to improve gases solubility in aqueous solutions. For instance, Yeom and Daugulis (2001) studied the use of n-hexadecane (HEX) as an absorbent for benzene in a bubble column, achieving 90% removal. Also, Dumont et al. (2011) found that silicone oil (SO) could improve the gas–liquid mass transfer for hydrophobic volatile organic compounds (VOCs) and be integrated in a process of absorption and biological reduction. Muñoz et al. (2008) achieved 12-fold higher elimination capacities (EC) and removal efficiencies (RE) when using an organic phase (2,2,4,4,6,8,8-heptamethylnonane (HNO)) in the removal of a hydrophobic VOC (alpha pinene). Furthermore, Yu and Munasinghe (2018) enhanced the gas fermentation process adding HEX as MTV to improve the gas–liquid mass transfer of carbon dioxide and oxygen.

Regarding the use of MTV for gaseous nitrogen species, the ability of SO as MTV for the enhancement of nitrous oxide (N_2_O) removal was described by Frutos (2018) in denitrifying batch assays using a *Paracoccus denitrificans* culture as inoculum. However, the author concluded that no significant enhancement was observed in N_2_O removal regardless of the amount of SO added. To the best of our knowledge, there is no evidence in the literature that non-ionic NAPs have been studied to enhance the mass transfer of NO.

Thus, this study aims to assess the feasibility of some NAPs (HEX, HNO, HTX, DSE, and SO) as MTVs to improve the mass transfer of NO to a liquid phase for further treatment with biological processes. To this aim, the multiphasic mechanisms of NAPs in two-phase system (gas/NAP) and in the three-phase system (gas/aqueous/NAP) were studied. Toxicity and biodegradability were investigated using heterotrophic denitrifying bacteria. CABR tests were performed, demonstrating the enhanced effect of using an MTV on NO reduction. This paper also describes a gas-to-liquid conversion pathway of NO using NAPs, including a description of possible process reactions and interactions with other nitrogen species that may be present in a CABR system.

## Materials and methods

### Microorganisms’ cultivation

Denitrifying biomass used in this study was cultivated in an 8 L sequential batch reactor (SBR) inoculated with biomass from the anoxic reactor of a municipal wastewater treatment plant (MWWTP) located in Manresa, Spain. The SBR was configured to develop 12-h cycles including 13 min of filling, 11 h and 15 min of reaction, 30 min of settling, and 2 min of withdrawal. The reactor had a pH control set at 8 using 1 M HCl addition. The reactor was fed with the solution described by Wang et al. (2019): 0.8 g L^−1^ NaNO_2_, 1.2 g L^−1^ C_2_H_3_NaO_2_·3H_2_O, 0.016 g L^−1^ KH_2_PO_4_, 0.041 g L^−1^ CaCl_2_, and 1-mL micronutrients solution (0.15 g L^−1^ H_3_BO_3_, 0.03 g L^−1^ CuCl_2_·2H_2_O, 0.18 g L^−1^ KI, 0.12 g L^−1^ MnCl_2_·4H_2_O, 0.06 g L^−1^ NaMoO_4·_2H_2_O, 0.12 g L^−1^ ZnSO_4_·7H_2_O, 0.15 g L^−1^ CoCl_2_·6H_2_O, and 10 g L^−1^ EDTA.Na_2_O_8_·2H_2_O). At steady state, the reactor was processing 0.087 g ${\mathrm{NO}}_2^{-}$ g ^−1^ VSS h^−1^.

### Mass transfer tests

To perform the mass transfer tests, the following NAPs were used: HEX (assay 99%; CAS: 544-76-3), DSE (assay 98%; CAS:110-40-7), HTX (assay 97%; CAS: 1873-88-7), HNO (assay 98%; CAS: 4390-04-9), and SO (assay 97%, CAS: 63148-52-7)). The NAPs were purchased from Sigma Aldrich (Lyon, France). NO (20% in nitrogen) and nitrogen (N_2_) were purchased from Linde Gas España (Rubí, Catalonia, Spain).

Two tests were performed to determine the mass transfer rate in the presence of NAP for the absorption of NO: (a) two-phase system (gas/NAP) and (b) three-phase system (gas/aqueous/NAP). Two-phase tests were performed in 120-mL glass amber bottles with an initial concentration of NO between 4500 and 5100 ppm_v_ in an inert atmosphere and different amounts of each of the tested NAPs. The blanks of these tests (two-phase system) were prepared with deionized water instead of NAP. Three-phase (gas/aqueous/NAP) tests were carried out in 500-mL glass amber bottles. The tests (three-phase system) consisted in a gas phase with an initial NO concentration between 4500 and 6000 ppm_v_ in an inert (nitrogen) atmosphere, an aqueous phase (deionized water or phosphate buffer at pH 8), and an organic phase (NAP). The ratio of NAP/aqueous solution tested was 5, 10, and 20%v/v. These tests had also a blank in which the NAP phase was replaced by an aqueous solution (deionized water or buffer).

The duration of the tests was 1 h, since preliminary studies found that the phase equilibrium within the system was reached in less than 1 h. Experiment monitoring was performed at the beginning and end of each test. All experiments were carried out per at least triplicate at a temperature of 25°C and 100 rpm orbital shaking in an incubator.

### Toxicity and biodegradability tests

Toxicity and short-term biodegradability tests were performed using an AER-500 respirometer (Challenge technology®) with an anaerobic configuration. The tests were carried out in 0.5-L glass bottles containing 0.3 L of denitrifying bacteria from the SBR at 0.95 g VSS L^−1^ in endogenous conditions. The production of gas in the form of N_2_ was measured to monitor the denitrifying activity of the bacteria, and thus to study the biodegradability and/or toxicity of the NAPs. Each of the tests was performed in duplicate including a control without NAP.

Specifically, toxicity tests were performed for each of the NAPs by injecting 10 mL of a solution containing 15.2 g L^−1^ sodium nitrite $\left({\mathrm{NaNO}}_2^{-}\right)$ and 26 g L^−1^ sodium acetate (C_2_H_3_NaO_2_·3H_2_O) as a carbon source. This is an excess of carbon source with respect to the denitrification stoichiometry as described in Eq.1 (Rittman and McCarty 2000). After the solution was injected, and after reaching a steady N_2_ production by the denitrifying bacteria, 50 mL of the tested NAP were injected into the bottle. The change in the N_2_ production rate indicated any potential inhibition occurring.

1 $$0.125{\mathrm{C}\mathrm{H}}_3{\mathrm{C}\mathrm{O}\mathrm{O}}^{-}+0.1358{\mathrm{N}\mathrm{O}}_2^{-}+0.744{\mathrm{H}}^{+}\overset{\mathrm{bacteria}}{\to }\ 0.0256{\mathrm{C}}_5{\mathrm{H}}_7{\mathrm{O}}_2\mathrm{N}+0.125{\mathrm{H}\mathrm{CO}}_3^{-}+0.345{\mathrm{H}}_2\mathrm{O}+0.055{\mathrm{N}}_2+0.004{\mathrm{C}\mathrm{O}}_2$$

Short-term biodegradability tests were performed by injecting 5 mL of a solution containing 15.2 g L^−1^ ${\mathrm{NaNO}}_2^{-}$ and 13 g L^−1^ C_2_H_3_NaO_2_·3H_2_O as limited carbon source (less than the stoichiometric amount with respect to nitrite). When the N_2_ production rate changed due to the lack of carbon source, 10 mL of the tested NAPs were added. A positive control was also used in which the carbon source was injected instead of NAP, to confirm that the bacteria were not inhibited. The evolution of the N_2_ production was monitored for 6 h.

Also, the ultimate biochemical oxygen demand (BOD) was tested for each NAP. Tests (long-term biodegradability) were performed with an OxiDirect® BOD measuring device and according to what is described in the OECD guide for testing chemical products (OECD 1992) using the respirometry manometric test 301F. The oxygen consumption due to pressure changes over 28 days was measured. The tests were carried out in 500-mL glass bottles using 1 mL of aerobic biomass from the Manresa WWTP at a final solid’s concentration of 7 mg TSS L^−1^ and 0.16 L of mineral medium (composition can be found in the OECD guide). The theoretical oxygen demand (thOD) of each of the compounds was calculated from the chemical formula through oxidation reaction, the following values were obtained: 0.23 mg O_2_ mg^−1^ HEX, 1.49 mg O_2_·mg^−1^ DES, 1.66 mg O_2_ mg^−1^ HTX, 0.23 mg O_2_ mg^−1^ HNO, 0.39 mg O_2_ mg^−1^ SO. The amount used of each NAP was: 175 mg HEX, 66 mg DES, 88 mg HTX, 4169 mg HNO, and 204 mg SO to provide the required COD (OECD guide).

### Chemical absorption and biological reduction—CABR tests

The tests were performed with the three most promising MTV based on the abiotic and biotic test results. CABR batch tests (four replicates per test) were performed in 120-mL glass amber vials previously filled with N_2_. Experimental conditions were a temperature of 25°C and orbital shaking of 100 rpm. Initial NO concentrations between 4500 and 6000 ppm_v_ were used. Two tests were performed varying the ratio between biomass and NAP. In the first test, 20 mL of biomass with a concentration of 1.09 g L^−1^ were used, followed by 2.5 mL of HTX, HNO, or HEX. In the second test, the biomass concentration was increased to 1.9 g L^−1^ while the NAP volume was maintained at 2.5 mL. All experiments had a blank containing neither NAP nor biomass and a control containing only biomass. The concentrations of NO and NO_2_ in the gas phase were measured at the beginning, 30 min and at the end of each test (1 h). Also, the liquid phase was monitored for pH, ${\mathrm{NO}}_2^{-}$ and ${\mathrm{NO}}_3^{-}$ concentration at the end of each test.

A gas phase kinetic study was performed by taking one of the replicates of the tests described above. The gas phase was analyzed at the beginning of the test and every 30 min for 4 h, then each vial was left shaking at 100 rpm at 25°C until 24 h had elapsed since the beginning of the experiment and finally the gas and liquid phases were analyzed again.

### Analyses

#### Chemical analysis

NO and NO_2_ concentrations in the gas phase were measured by Fourier transform infrared spectroscopy (FTIR) (PerkinElmer Inc., Spain), from an aliquot of 1 mL withdrawn from the test vials/bottles with a gas syringe (Dorhout et al. 2020). The NO removal efficiency (RE) in the tests was calculated from the difference in NO concentration at the beginning and end of the experiments.

Regarding the liquid phase, nitrogen compounds and chemical oxygen demand (COD) were analyzed in liquid samples after centrifuging at 15,000 rpm and filtration (0.22 μm) to remove NAPs and biomass. Concentrations of ${\mathrm{NO}}_2^{-}$ and ${\mathrm{NO}}_3^{-}$were analyzed using Hach Lange kits LCK 342 and LCK 339 (Germany). The LCK 342 kit uses the diazotization method with a measurement range of 0.6-6.0 mg L^−1^ for ${\mathrm{NO}}_2^{-}-N$. The LCK 339 kit employs the 2,6-dimethylphenol method, with a measurement range of 0.23–13.5 mg L^−1^ for ${\mathrm{NO}}_3^{-}$-N. COD was quantified using Hach Lange kits LCK 114 and LCK 214 (Germany). These kits employ the dichromate method and have measurement ranges of 150–1000 mg L^−1^ O_2_ for LCK 114 and 0-1000 mg L^–1^ O_2_ for LCK 214. All kits used in the study were compliant with the relevant ISO and DIN standards.

#### Microbial community analysis

Microbial composition of the biomass used for the study was analyzed. DNA extraction from a biomass sample was performed using the DNeasy PowerSoil Pro Kit (Qiagen, German) according to its principles and instructions and stored at −70°C. Two variable regions (V3, V4) of the 16S rRNA gene were amplified by polymerase chain reaction (PCR) with custom designed fusion primers described by Torrell et al. (2021). Bovine serum albumin (BSA) was added to the PCR reaction to neutralize potential inhibitors. The PCR product, called amplicon or library, was visualized with a 2% agarose gel, purified with the NucleoSpin kit (Macherey-Nagel, Berlin, Germany), and quantified with an Agilent 2100 Bioanalyzer (Agilent Technologies, California, USA) and the Agilent High Sensitivity DNA kit (Agilent Technologies). Lastly, an equimolar mixture (60pM) of the samples was created and the Ion 520 and Ion 530 Kit-Chef (Life Technologies, Carlsbad, California, USA) and a 530 chip were used for the sequencing on LifeTechnologies GeneStudio S5 machine, using 850 flows per run. The obtained data was analyzed using the software QIIME 2–2020.8.

#### Denitrifying capacity—qPCR and gene expression

The same sample used to study the microbial community was used to study denitrification genes expression (nirS, nosZ, and CnorB). For this purpose, RNA was extracted with the Rneasy PowerSoil Total RNA Kit (Qiagen, German) according to its principles and instructions and stored at −80°C until analysis. From the set of primers published by Razaviarani et al. (2019), three pairs of primers were synthesized to selectively amplify the nirS, cnorB, and nosZ genes. A block for each of the genes (synthetic DNA sequence containing the target) served as a positive control and standard for quantification. RNA samples were retrotranscribed with a commercial polymerase (Super script IV (Invitrogen)) and assayed by real-time quantitative PCR for all 3 genes, using the Power-up sybr green master mix (Thermofisher) and the Applied biosystems 7900HT quantitative PCR equip. It should be noted that the primers analyzed where those corresponding to the denitrification steps from ${\mathrm{NO}}_2^{-}$ because this was the nitrogen source in the SBR used for biomass cultivation.

## Results and discussion

### Mass transfer from gas-to-liquid of nitric oxide in a two-phase system

**Fig. 1:**
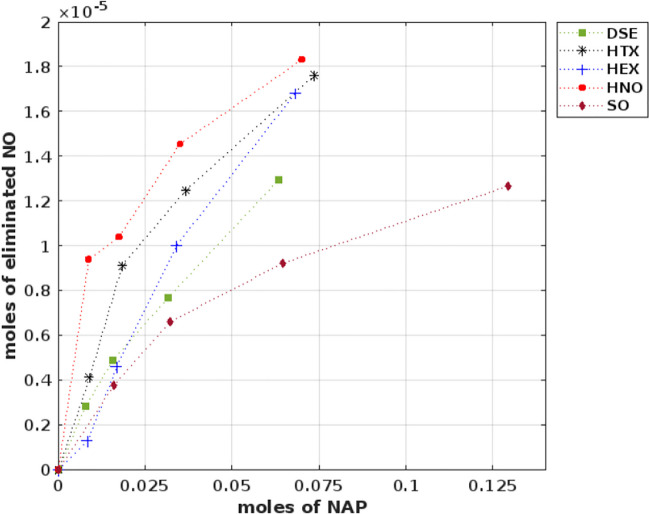
Ratio of NO removal vs. amount of mass transfer vector (NAP) in a two-phase system

Figure 1 shows the decrease of NO concentration in the gas phase using SO, DSE, HTX, HNO, and HEX as MTV at different amounts of NAP in the two-phase system. It was found that the increase in the amount of NAP produced a greater removal of NO in the gas phase. In addition, from the slope of the linear regression, the maximum capacity of mass transfer in the two-phase system was calculated, which is reported in Table 1. Results showed that HNO had the maximum mass transfer capacity with 0.32 mol NO/kmol NAP, followed by HTX with 0.29 mol NO/kmol NAP, HEX 0.26 mol NO/kmol NAP and DSE 0.22 mol NO/kmol NAPs. These results are consistent with those described by Dumont (2019) where he indicates that an increase (up to tens of times) in the amount of NAP helps to improve mass transfer for compounds that are very poorly soluble in water as is the case for NO, while for other gases that are moderately insoluble in water the use of NAP is less significant. All NAPS resulted in a similar mass transfer gas-to-liquid capacity except for SO (0.09 mol NO/kmol NAP). This agrees with what was found by Frutos (2018) who did not find significant improvements in the reduction of N_2_O using SO as MTV.

**Table 1:** Linear regression slopes and maximum mass transfer gas-to-liquid capacity of two-phase systems

	Slope	*r*^2^	mol NO/kmol NAP)
HEX	2.55E-04	0.99	0.26
HTX	2.88E-04	0.94	0.29
HNO	3.16E-04	0.87	0.32
DSE	2.18E-04	0.98	0.22
SO	1.21E-04	0.94	0.12

FTIR spectra of nitrogenous species in the gas phase (supplementary information Figure S1) indicated that HTX and HEX the MTV were capable to oxidize NO to NO_2_, thus evolving from a slightly soluble compound to a more soluble one. It is important to note that NO has a high sensitivity to oxygen, which makes the chemical reactions that occur in a two-phase system (gas/NAP) not comparable to other gases such as VOCs, CO_2_, and H_2_S. However, there was no evidence of oxidation for DSE; in this case, although there was a decrease in NO concentration in the gas phase, the formation of NO_2_ was not evident in the FTIR spectrum.

### Mass transfer from gas-to-liquid of nitric oxide in a three-phase system

**Fig. 2:**
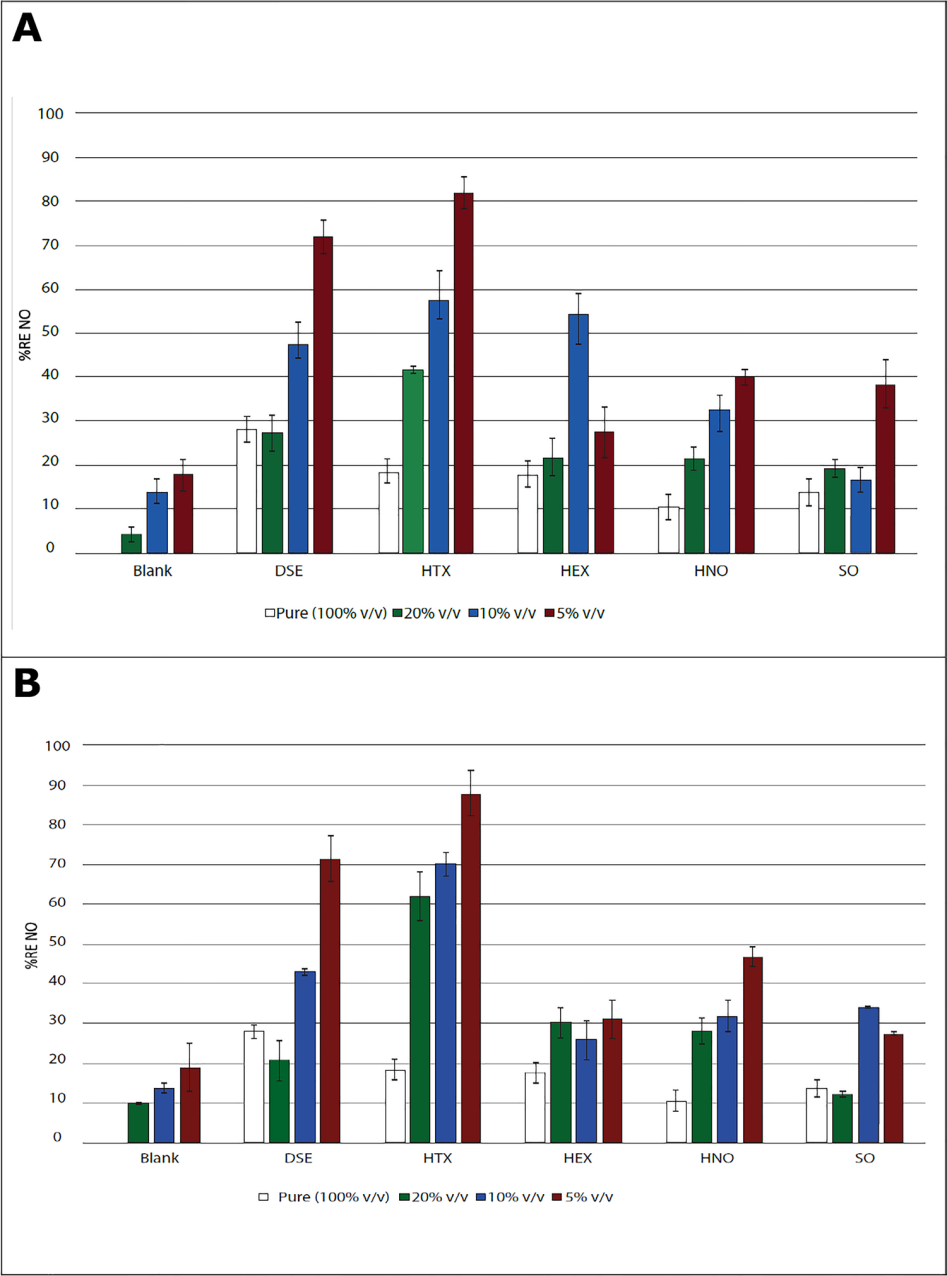
Mass transfer tests in a three-phase system. **A** NAP+ water and **B** NAP + phosphate buffer—v/v indicates the volume of NAP in the volume of the total liquid

Figure 2 shows the RE of NO using HEX, HNO, HTX, SO, and DSE in three-phase system (gas/aqueous/NAP) containing water (A) or phosphate buffer (B) as aqueous solutions. The NAPs that gave the best results in terms of percentage of removed NO in the gas phase were HTX (82 ± 3% in water; 88 ± 6% in buffer, DSE (72 ± 4% in water; 71 ± 6% in buffer), and HNO (40 ± 2% in water; 47 ± 3% in buffer) with 5% v/v of each NAP. From these values, it can be concluded that no significant difference was observed whether buffer or water was used. Additionally, it was observed (also in Figure 2) that in most of the NAPs, the mass transfer of NO was improved when an aqueous liquid phase was put in contact with the NAP in comparison with the NAPs in its pure state. This is an advantage because biological processes take place in aqueous media despite some authors point out that there may also be bacterial activity in the organic phase (Lebrero et al. 2019).

It is worth mentioning that a decrease in pH from approximately 6 to 3 was observed due to the mass transfer of NO when water was used. This is basically because NO is an acid gas, whereas the pH remained at 8 when phosphate solution was used due to its buffer potential effect. This is consistent with what was observed by Guimerà et al. (2021), where no gas-to-liquid mass transfer was observed when a phosphate buffer was added in a system without NAP. However, the system was kept at a neutral pH.

**Fig. 3:**
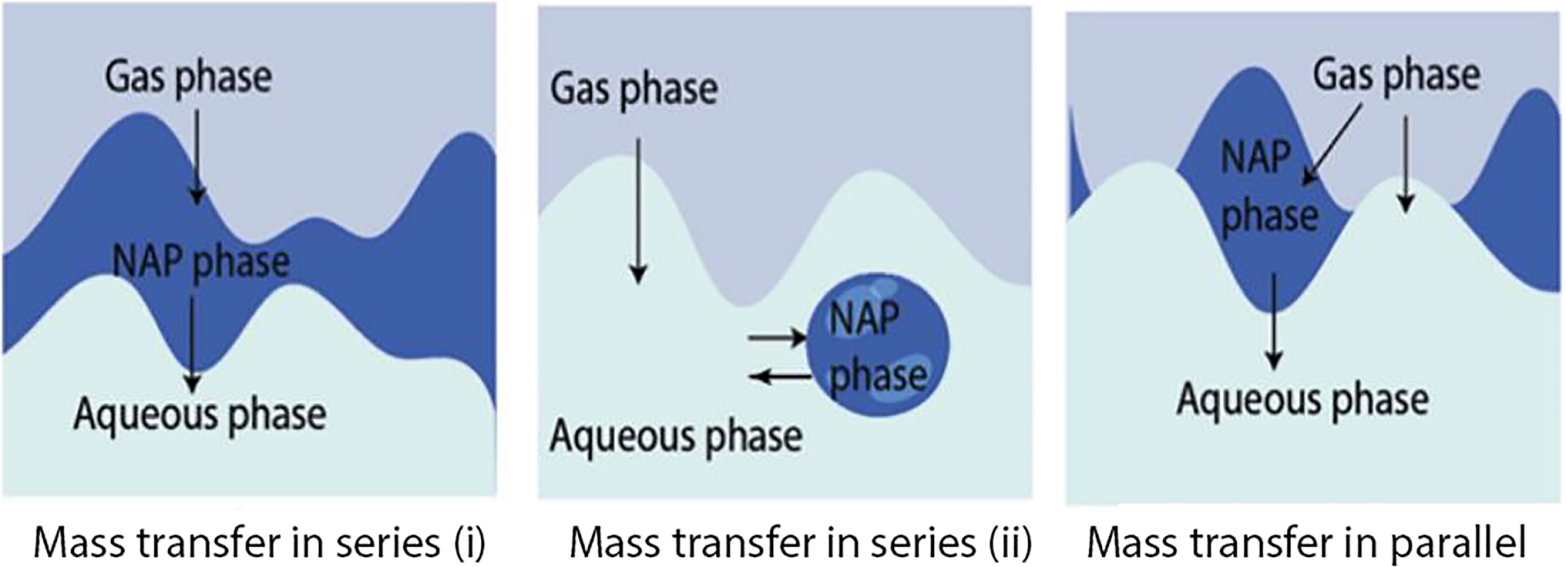
Possible pathways for mass transfer in the aqueous phase (adapted by Dumont and Andrès 2012)

Regarding the phenomenon of mass transport in a three-phase system, several studies of VOCs indicate that it can occur in two different ways: in series and in parallel. Related to the “in series” pathway, Figure 3 indicates that it can occur in two ways:

(i) Gas phase contacting the NAP, being a vector for gas-to-liquid mass transfer to the aqueous phase to take place
(ii) Gas phase contacting the aqueous phase (gas-to-liquid mass transfer) without direct contact of the gas with the NAP

The gas-to-liquid mass transfer can also occur “in parallel,” as there can be a simultaneous contact between the three phases (gas, NAP, and aqueous phase) together or separately (Dumont and Andrès 2012). According to the results of the experiments performed (two-phase and three-phase systems), neither of these two mass transfer pathways can be identified. However, according to the latest findings of Dumont (2019) with VOCs, it indicates that mass transfer does not occur “in parallel,” being “in series” the most likely pathway.

A literature survey of NAPs as MTV to improve gas-to-liquid mass transfer of NO showed that only organic tertiary hydroperoxides in HEX solution were studied as oxidants to improve NO solubility (Perlmutter et al. 1993). The other NAPs (DSE, HNO, HTX, SO) that were tested in this study were not previously studied with NO. This being the first study where the feasibility of using NAP to improve the absorption of a very hydrophobic gas such as NO is analyzed.

Aqueous liquid phase analysis (supplementary information Figure S2) showed a higher concentration of ${\mathrm{NO}}_2^{-}$ and ${\mathrm{NO}}_3^{-}$ at the end of the tests when the proportion of NAP in the liquid was higher. For example, a ${\mathrm{NO}}_3^{-}$ concentration of 155.75 ± 19.45 mg L^−1^ (water) and 167.33 ± 11.51 mg L^−1^ (phosphate buffer) was found when there was 20% v/v of HTX in the liquid while only a ${\mathrm{NO}}_3^{-}$ concentration of 50.50 ± 10.58 mg L^−1^ (water) and 36.83 ± 11.45 mg L^−1^ (phosphate buffer) was found with 5% v/v of HTX in the liquid.

According to supplementary information Figure S2, the results confirmed that as soluble gases (NO_2_) were formed in the gas phase, they were transferred to the liquid phase through different instantaneous and irreversible reactions with water or phosphate buffer to form nitrous and nitric acids as described by Guimerà et al. (2021). The results of the mass transfer in the three-phase system (Figure S2) showed a higher concentration of ${\mathrm{NO}}_2^{-}$ and ${\mathrm{NO}}_3^{-}$ in the aqueous phase when MTV was present compared to NAP-free blanks. This confirmed that the gas-to-liquid NO transfer was improved by the tested MTVs. Specifically, it can be observed in Figure S2 (A) that there was a higher concentration of ${\mathrm{NO}}_3^{-}$ than ${\mathrm{NO}}_2^{-}$in the aqueous phase when the test was carried out with water. However, with phosphate buffer (Figure S2 (B)) the concentrations of ${\mathrm{NO}}_3^{-}$ and ${\mathrm{NO}}_2^{-}$ were similar. Therefore, as described by Thomas and Vanderschnren (2000), when only water was added as the aqueous phase, the system favored ${\mathrm{NO}}_2^{-}\kern0.5em$production due to the reaction of HNO_2_ and N_2_O_3_. However, when using a phosphate buffer, it also generated ${\mathrm{NO}}_3^{-}$, which is formed due to the absorption and reaction of NO_2_ and N_2_O_4_.

### Toxicity and biodegradation test with denitrifying bacteria

**Fig. 4:**
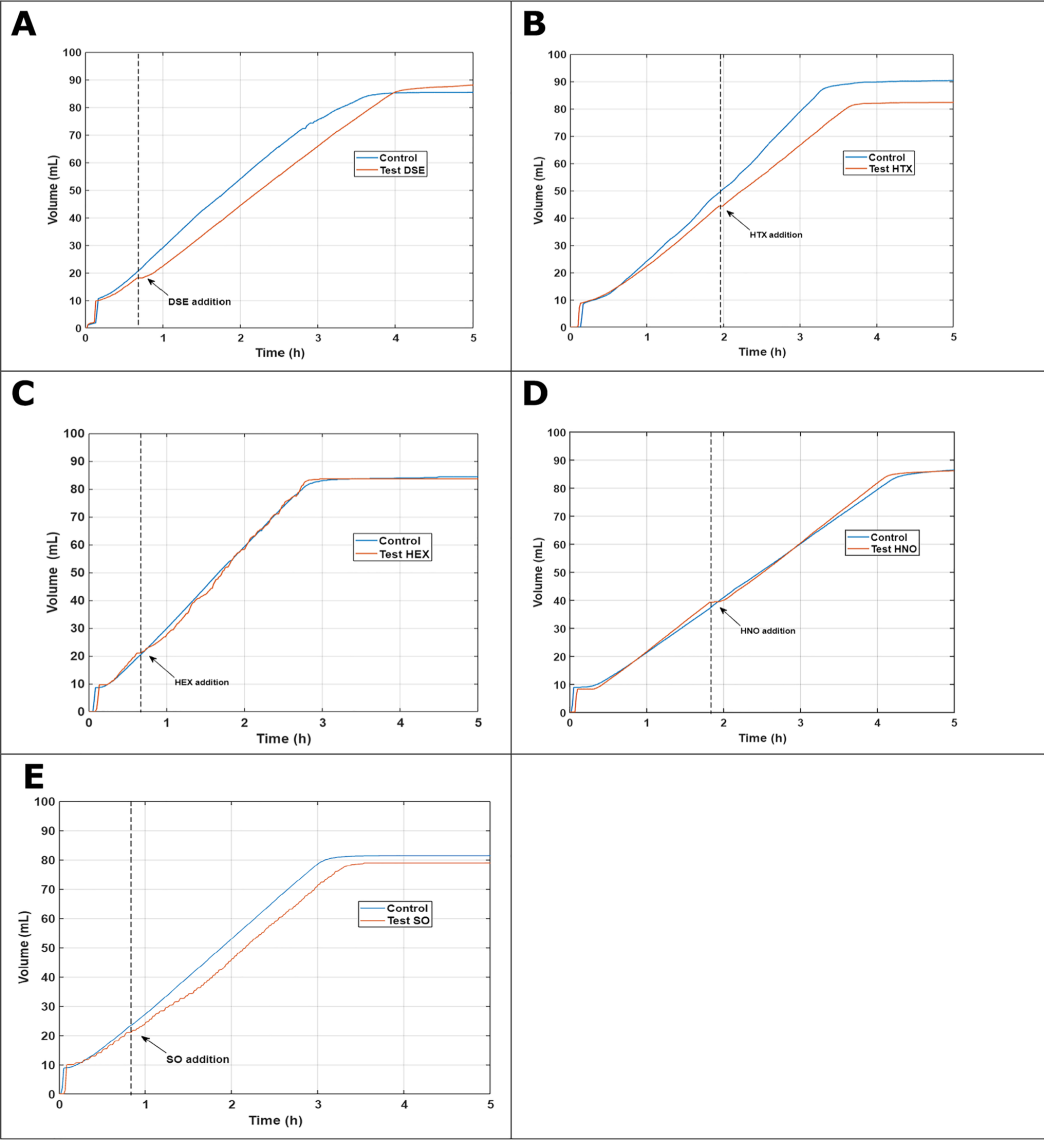
Toxicity test through denitrifying bacteria—short term. **A** DSE, **B** HTX, **C** HEX, **D** HNO, **E** SO

Toxicity tests were performed to assess whether these compounds could affect the biological activity of denitrifying bacteria. Results demonstrated that none of the NAPs were toxic for denitrifying bacteria at the short-term (see Figure 4), since there was no significant change in the slopes of each activity test after adding any of the NAPs. These results are consistent with other studies that tested specific denitrifying strains; for instance, Köhler et al. (1994) studied the degradation of phenanthrene by *Pseudomonas aeruginosa* AK1 using HNO as a MTV, confirming that HNO improves the solubility of anthracene without being toxic to AK1. Also, Marcoux et al. (2000) studied the degradation of high-molecular-weight polycyclic aromatic hydrocarbons (HMW PAHs) such as pyrene, chrysene, benzo[a]pyrene and perylene with SO, HNO, and HEX as MTVs using the microorganism *Pseudomonas aeruginosa* 57RP and they verified that the denitrifying bacteria did not present toxicity in the presence of these MTVs.

The importance of using non-biodegradable MTVs should be emphasized when thinking on industrial application of these compounds in continuous NO removal systems. If MTVs were biodegradable, they would need to be supplied continuously, which would increase the operating costs of the process. Short-term biodegradability tests showed that all NAPs, except DSE, had no apparent biodegradability under the tested conditions (supplementary information Figure S3). Long-term tests showed a biodegradation of 0% for SO, 1% for HNO, 7% for HTX, 8% for HEX, and 100% for DSE. The tests also confirmed that DSE was completely biodegradable under both anoxic and aerobic conditions. The biodegradability of the NAPs was also tested by Muñoz et al. (2008) using the Zahn-Wellens-EMPA test. They showed that SO, HEX, HNO, and HTX are biocompatible and non-biodegradable and DSE was mineralized specifically with the denitrifying bacterium *Pseudomonas fluorescens* NCIMB 11671, which supports the results of the present study. As the toxicity and biodegradability of these compounds with a mixed culture of denitrifying bacteria have not been previously studied, these results will serve as a guide for future applications in the industry.

### Chemical absorption and biological reduction—CABR tests

**Fig. 5:**
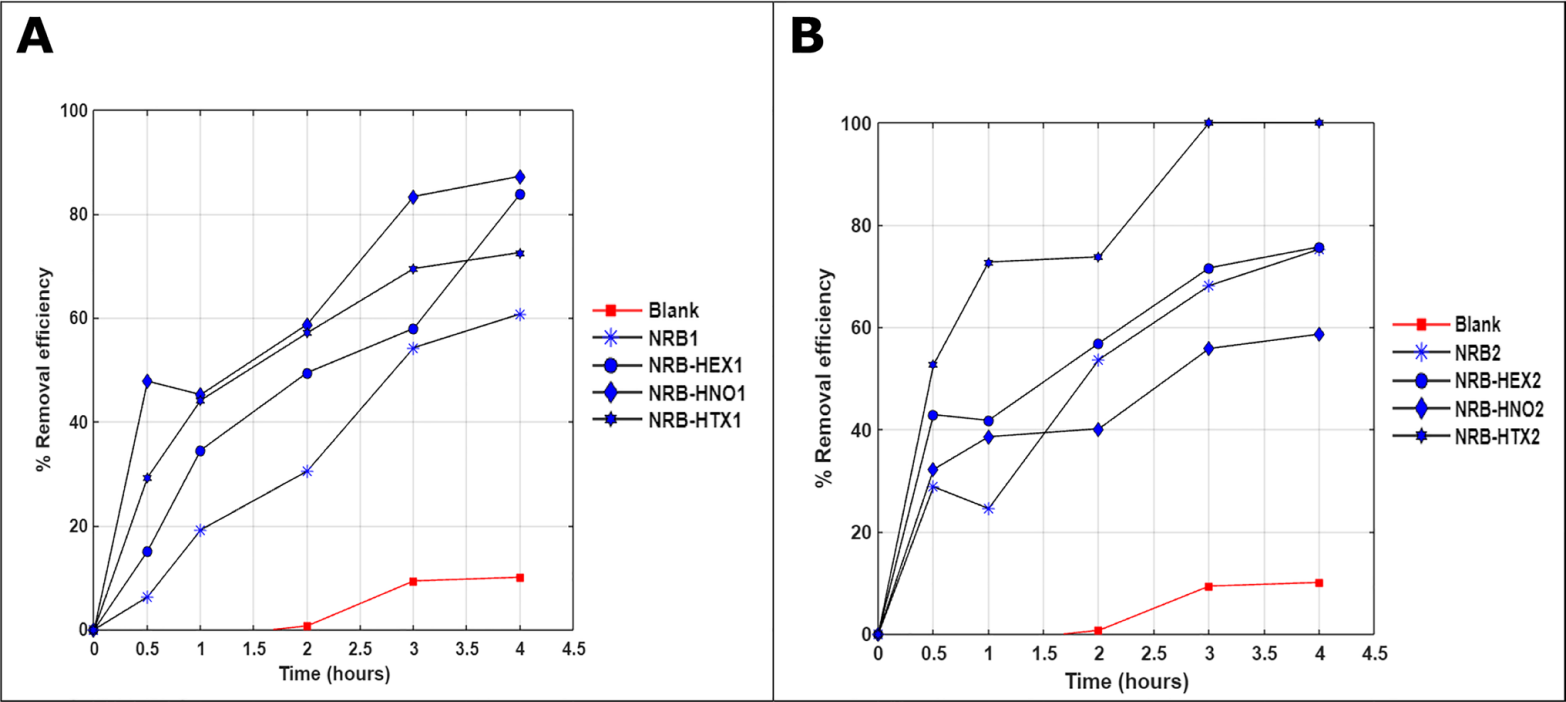
Removal efficiency of NO with NRB with a concentration: **A**1.09 g VSS L^−1^ and **B** 1.88 g VSS L^−1^ and 2.5 mL of NAP

HEX, HNO, and HTX were chosen to study the integration of the chemical absorption and biological reduction processes, because these MTVs demonstrated high absorption rates, no significant toxicity, and almost no biodegradability. These are some of the indicators recommended by Bruce and Daugulis (1991) to choose a suitable MTV. The results showed an improvement in NO abatement by adding any of the three MTVs tested and increasing the biomass concentration of denitrifying bacteria in the system. This is verified in the kinetics of the NO removal in the gas phase in the CABR test during the first 30 min (Figure 5). Biomass with no NAP (1.09 g VSS L^−1^) showed only a RE NO of 6%. Adding the NAPs, a RE NO of 48% was reached using HNO, 29% with HTX and 15% with HEX. When the biomass concentration was increased (1.88 g VSS L^−1^), the NO RE of the positive control without NAP improved up to 29%. However, there were better results when NAPs were used (HTX: 53% RE NO; HEX: 35% RE NO; HNO: 32% RE NO).

The results of the kinetics in the gas phase at 24 h showed 100% NO removal when MTV was and was not added to the system. However, in these systems, the removal in the first minutes (30 min) is the most important in case a bioreactor is to be sized in the future. In addition, since it is a system with biological elimination, it is normal and to be expected that when there is a higher concentration of biomass, the system will go faster. This also indicates that there is biological elimination.

**Fig. 6:**
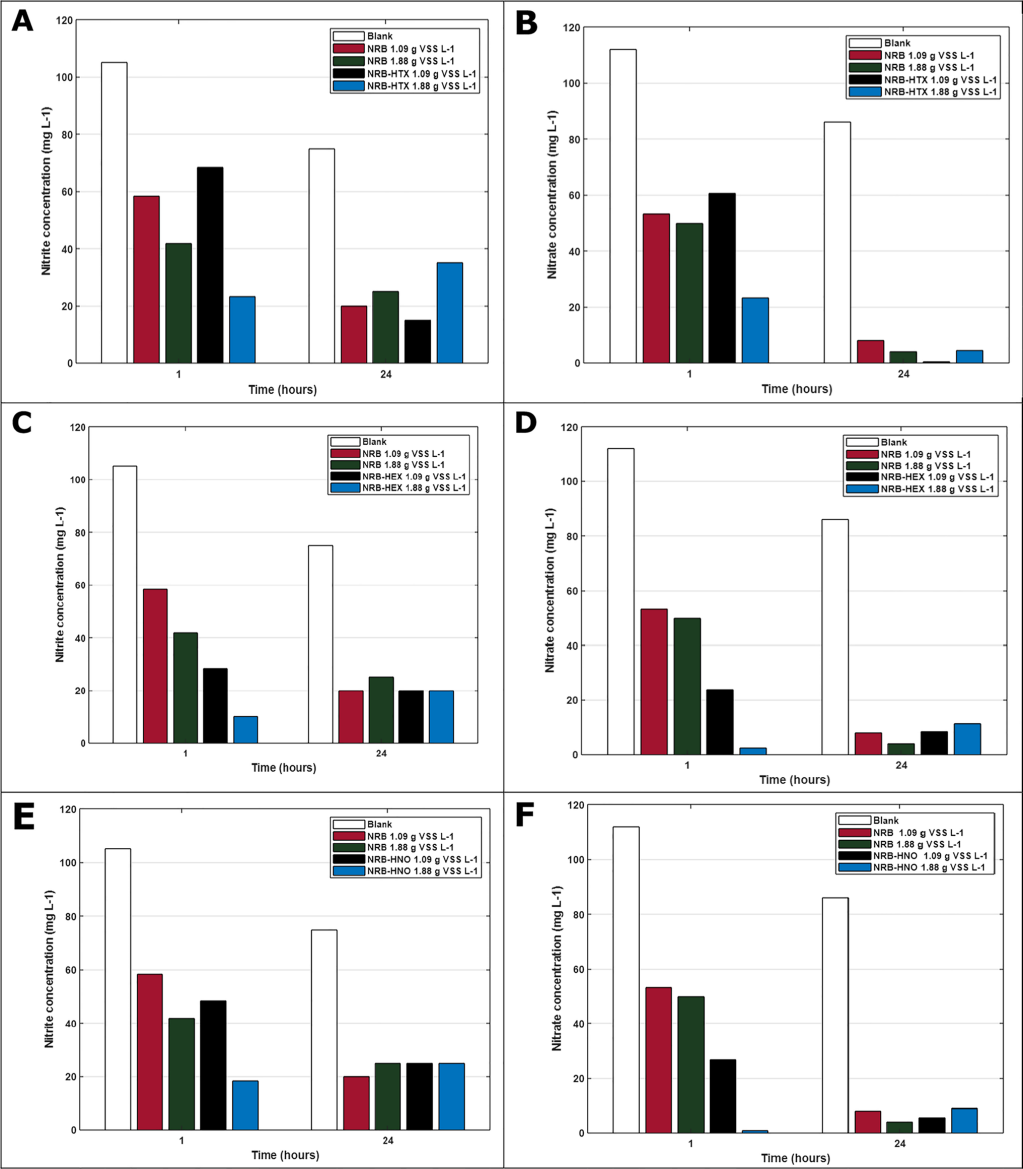
Concentration of ${\mathrm{NO}}_2^{-}$ and ${\mathrm{NO}}_3^{-}$ in the CABR experiments at 1 h and 24 h: **A**–**B** HTX; **C**–**D** HEX; **E**–**F** HNO

The concentration of ${\mathrm{NO}}_3^{-}$ in the liquid phase (Figure 6) showed some accumulation of ${\mathrm{NO}}_3^{-}$ during the first hour that was almost fully removed at 24 h, which indicates that denitrification was taking place in the system. However, for ${\mathrm{NO}}_2^{-}$ after 24 h there was still some accumulation. This is because the ${\mathrm{NO}}_2^{-}$ present in the system is produced both by the oxidation process of the NO, NO_2_ N_2_O_3_, and N_2_O_4_ and the denitrification step from ${\mathrm{NO}}_3^{-}$ to ${\mathrm{NO}}_2^{-}$(Suchak et al. 1990).

There are many studies where different NAPs (e.g., SO, DSE, HNO, HTX) were used to reduce hydrophobic VOCs compounds (Quijano et al. 2009; Darracq et al. 2010). However, in terms of NO, the chemistry and the absorption/oxidation reactions are completely different than VOCs because there are many nitrogen species involved (NO_2_, N_2_O, N_2_O_3_, N_2_O_4_), that make understanding mass transfer (gas/aqueous phase/NAP) more complex.

**Fig. 7:**
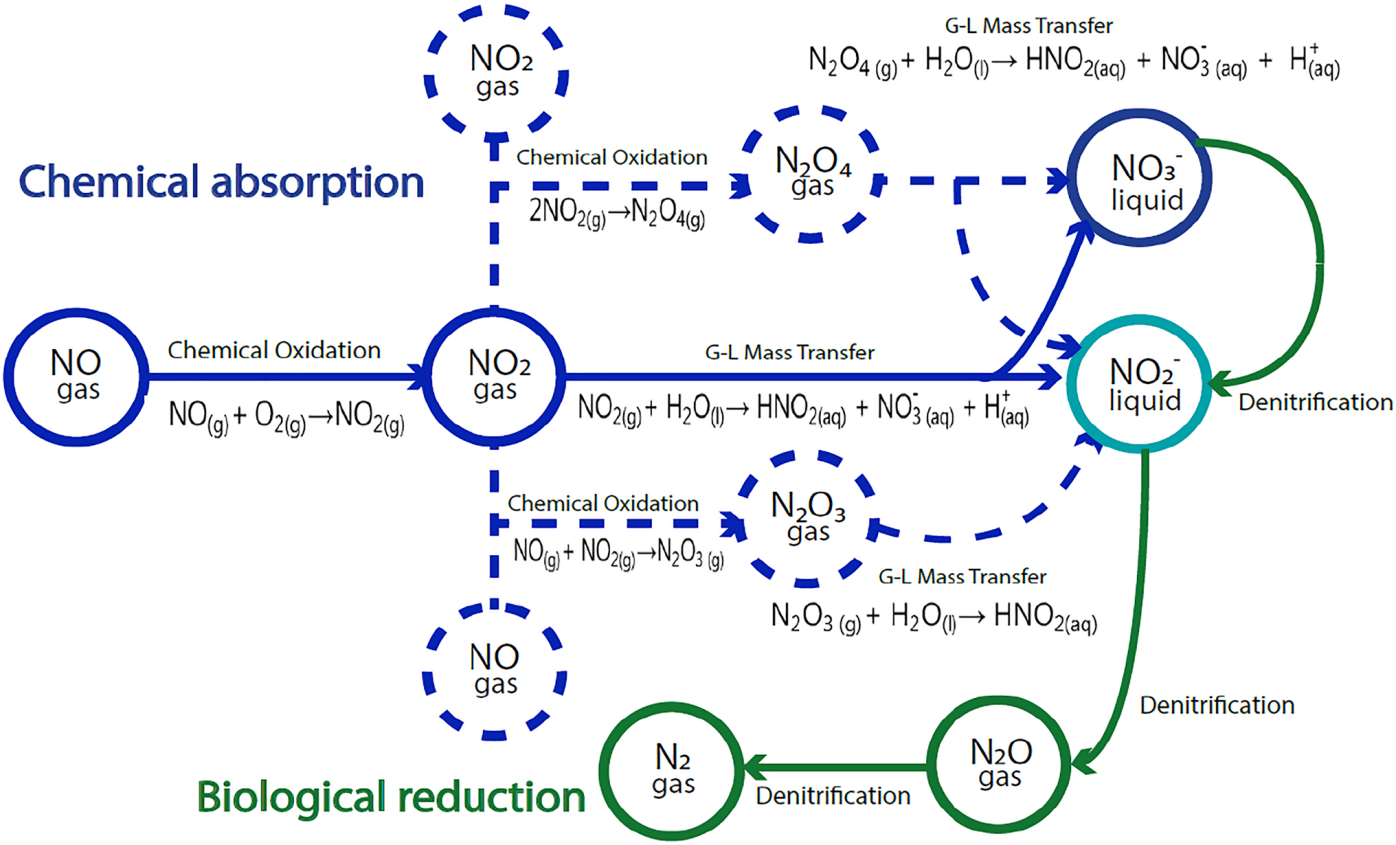
Proposed CABR pathway for NO

Figure 7 shows a proposed NO conversion pathway in a system using NAP as MTV. In the gas phase, in a first stage an oxidation reaction of NO to NO_2_ occurs in the presence of O_2_ and MTV. In addition to the oxidation reaction, NO and NO_2_ can form other nitrogen species such as dinitrogen trioxide (N_2_O_3_) and dinitrogen tetroxide (N_2_O_4_) that have different oxidation states and are more soluble than NO. Once all nitrogen species are transferred from the gas to the liquid phase, ${\mathrm{NO}}_2^{-}$ and ${\mathrm{NO}}_3^{-}$ are formed. In a last stage denitrifying bacteria consume this ${\mathrm{NO}}_2^{-}$ and ${\mathrm{NO}}_3^{-}$for their metabolic process and these species are converted first to N_2_O and then to N_2_.

As described above, the most used MTV for solubility enhancement has been Fe((II)EDTA^2−^. Comparing the NO conversion pathway using Fe(II)EDTA^2−^ and NAP, the main difference is that NAP promote oxidation of NO to NO_2_, while Fe(II)EDTA^2−^ is a chelating agent that binds to the NO molecule (Fe(II)EDTA-NO) and then serves as an electron donor for nitrite reducing bacteria (NRB) (Li et al. 2014). In addition, when Fe(II)EDTA is used as MTV, it was observed that N_2_O accumulation can occur if there are high concentrations of the complex Fe(II)EDTA-NO^2−^ (Chen et al. 2016). In contrast, in the case of NAP, no inhibitions were observed for high concentrations of NAP (MTV), mainly because they are not soluble, not toxic and do not affect the denitrification process.

### Microbial community analysis and qPCR

**Fig. 8:**
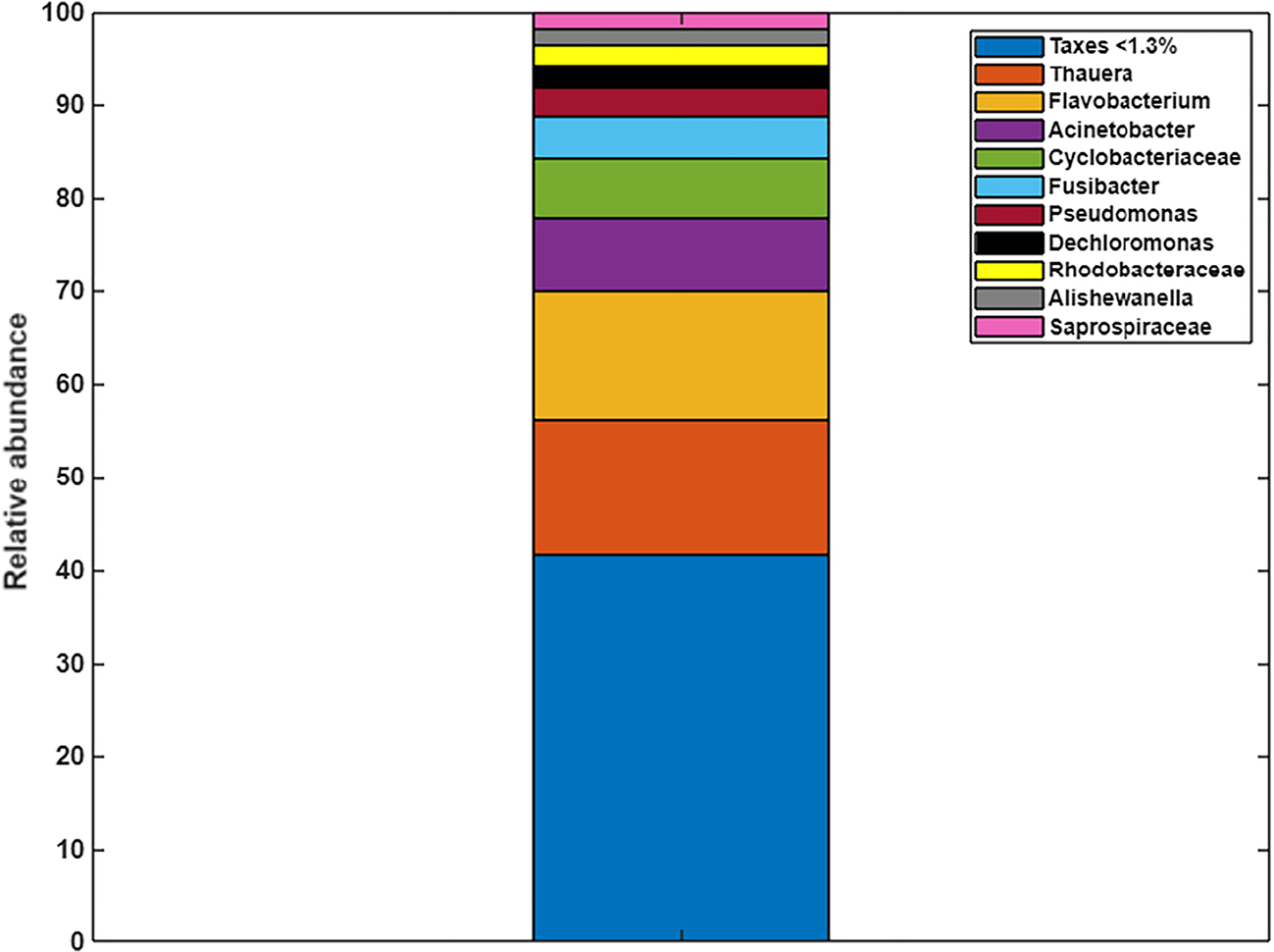
Microbial community structure (at genus level) in the biomass used for CABR experiments

The microbial community structure was analyzed to investigate the species existing in the biomass used for the CABR test. As shown in the Figure 8, at order genus, *Thauera* (14.37%), *Flavobacterium* (13.87%), *Acinetobacter* (7.90%), *Cyclobacteriacea* (6.41%), *Fusibacter* (4.41%), *Pseudomonas* (3.19%), *Dechloromonas* (2.40%), *Rhodobacteraceae* (2.25%), *Alishewanella* (1.75%), and *Saprospiraceae* (1.74%) were dominant. As shown in the relative abundance graph (Figure 8), the inoculum used in the CABR tests was mainly composed by different denitrifying species involved in different metabolic pathways, indicating that the biomass was fully conditioned for denitrification and reaching high RE NO. The benefit of having species diversity in denitrification processes was demonstrated by Patel et al. (2021) in which he found that genera such as *Pseudomonas*, *Thauera*, *Azoarcus* were positively correlated with each other and enhanced the denitrification process in a moving bed biofilm reactor (MBBR).

Previous studies have found similar bacterial communities involved in in denitrification processes. For instance, gram-negative bacteria such as facultative anaerobic (*Pseudomonas*), diazotrophic (*Rhodobacte*r), and pathogenic (*Flavobacterium*) have been reported previously in denitrifying processes (Zumft 1997). The genus *Acinetobacter* includes the most isolated denitrifying bacteria in WWTP (Knowles 2008). In addition, the genus *Thauera*, as a denitrifying group of species, has been detected in different systems with different treatment objectives. For example, Xu et al. (2018) detected it in an acetate-fed denitrifying system, Liang et al. (2020) in a denitrifying system using H_2_S as an electron donor, and Rabus and Widdel (1995) in a degrading alkylbenzenes system under denitrifying anaerobic conditions.

In denitrification processes, it is important to make sure that the complete process up to nitrogen gas is taking place avoiding the production of N_2_O, which is a greenhouse gas. Therefore, it is necessary to check for the presence or absence of the genes codifying the different transformation steps. The presence or absence of the different denitrifying genes was assessed by checking if these genes appear in the completed genomes of all the taxonomic categories that were found by the metagenomic approach. As shown in Table 2, presence of all three genes: nirS (${\mathrm{NO}}_2^{-}$ to NO), CnorB (NO to N_2_O) and nosZ (N_2_O to N_2_) was only found in *Pseudomonas*. Also, the most common gene found was nosZ, being *Thauera*, *Flavobacterium*, *Pannonibacter* the genera that were most abundant in the analyzed sample and were carrying this gene. Additionally, the genera *Streptococcus*, *Bacillus*, *Legionella*, *Streptomyces*, and *Corynebacterium* were found to have the presence of the CnorB gene.

**Table 2:** Presence/absence of nosZ, CnorB, and nirS into the genome of the identified genes. Percentage of abundance reflects the contribution of this genera to the overall sample of the biomass used in the experiments

Identified genus	Percentage of abundance in the sample	Presence of the gene in the genome		
		nirS ${\mathrm{NO}}_2^{-}\kern0.5em \to \mathrm{NO}$	CnorB NO → N_2_O	nosZ N_2_O → N_2_
*Thauera*	13.95%	Absent	Absent	Present
*Pannonibacter*	4.69%	Absent	Absent	Present
*Flavobacterium*	4.52%	Absent	Absent	Present
*Chryseobacterium*	0.80%	Absent	Absent	Present
*Pseudomonas*	0.41%	Present	Present	Present
*Rhodobacter*	0.29%	Absent	Absent	Present
*Hyphomicrobium*	0.20%	Absent	Absent	Present
*Paracoccus*	0.17%	Absent	Absent	Present
*Shinella*	0.14%	Absent	Absent	Present
*Mesorhizobium*	0.09%	Absent	Absent	Present
*Bacteroides*	0.06%	Absent	Absent	Present
*Bacillus*	0.05%	Absent	Present	Present
*Streptomyces*	0.04%	Absent	Present	Absent
*Prevotella*	0.03%	Absent	Absent	Present
*Bradyrhizobium*	0.03%	Absent	Absent	Present
*Legionella*	0.03%	Absent	Present	Absent
*Streptococcus*	0.02%	Absent	Present	Absent
*Castellaniella*	0.02%	Absent	Absent	Present
*Ensifer*	0.01%	Absent	Absent	Present
*Afipia*	0.01%	Absent	Absent	Present
*Elizabethkingia*	0.01%	Absent	Absent	Present
*Corynebacterium*	0.01%	Absent	Present	Absent
*Luteitalea*	0.01%	Absent	Absent	Present

The overall microbial composition can be determined from quantification of functional genes by DNA amplification. However, to capture the immediate microbial response, it is important to perform RNA quantification, and since nirS and nosZ have been reported to be good predictors of denitrification potential (Petersen et al. 2012), nirS, nosZ, and CnorB gene expression was assessed by qPCR. showed that. The results showed a clear expression of the nosZ gene, while the expression of nirS and CnorB was not positive. As a conclusion, it can be assumed that the denitrifying ability was mainly due to nosZ (N_2_O to N_2_), rather than the other 2 genes, and thus shows complete denitrification.

## Conclusions

The results of this work showed that HEX, HNO, HTX, DSE, and SO could be used as mass transfer vectors for CABR systems of NO, with HNO being the compound that showed the highest absorption capacity in a two-phase system (gas phase/NAP) system. Additionally, none of the NAPs studied turned out to be toxic for denitrifying bacteria. The CABR tests demonstrated the feasibility of the entire system with HEX, HNO, and HTX and the improvement of NO RE when using these MTVs. This is the first study to demonstrate the NO conversion pathway with its different reactions in the gas phase (oxidation), mass transfer from the gas phase to the liquid phase promoted by NAPs, and biological denitrification in the aqueous phase. Although this research is a first step, it is necessary to evaluate this process in a continuous system where all transport phenomena are studied in more detail.

### Supplementary information

ESM 1 (DOCX 769 kb)

## Data Availability

The authors declare that the data supporting the findings of this study are available within the paper and its Supplementary Information files. Should any raw data files be needed in another format they are available from the corresponding author upon reasonable request.
