# Supplementary material for: Mass transfer vectors for nitric oxide removal through biological treatments
Source: Environ Sci Pollut Res Int. 2023 Oct 2;30(51):110089–103. doi: 10.1007/s11356-023-30009-6 (PMC10625516; doi:10.1007/s11356-023-30009-6)
Supplement: Supplementary file 1 — (DOCX 769 kb) [file 11356_2023_30009_MOESM1_ESM.docx]

**MASS TRANSFER VECTORS FOR NITRIC OXIDE REMOVAL THROUGH BIOLOGICAL TREATMENTS**

**Environmental Science and Pollution Research**

David Fernando Cubides Páez^a-b^, Xavier Guimerà Villalba^b*^, Nerea Abasolo Zabalo^c^ Helena Torrell Galceran^c^, Irene Jubany Güell^a^, Xavier Gamisans Noguera^b^

^a^Eurecat, Centre Tecnològic de Catalunya, Sustainability Area, Manresa 08243, Spain

^b^Department of Mining, Industrial and ICT Engineering (EMIT), Biological Treatment of Gaseous Pollutants and Odours Group (BIOGAP), Manresa School of Engineering (EPSEM), Universitat Politècnica de Catalunya (UPC), Av. Bases de Manresa 61-73, 08242 Manresa, Spain

^c^Eurecat, Centre Tecnològic de Catalunya, Centre for Omic Sciences (COS), Joint Unit Universitat Rovira i Virgili-EURECAT, Unique Scientific and Technical Infrastructures (ICTS), 43204 Reus, Spain

***Corresponding author: Xavier Guimerà

E-mail: xavier.guimera@upc.edu

Tel:+34 [938 77 72 00](https://www.google.com/search?q=epsem+manresa&rlz=1C1GCEB_enES922ES922&sxsrf=ALiCzsagAcFszTDBAPOgy9AxDlPxoVBm1A%3A1653036078498&ei=LlSHYtqCHsj2lwSWuYqIBA&ved=0ahUKEwja0Ymi1-33AhVI-4UKHZacAkEQ4dUDCA4&uact=5&oq=epsem+manresa&gs_lcp=Cgdnd3Mtd2l6EAMyCwguEIAEEMcBEK8BMgIIJjoHCAAQRxCwAzoECCMQJzoKCC4QxwEQrwEQQzoECAAQQzoLCAAQgAQQsQMQgwE6CgguEMcBEK8BECc6EQguEIAEELEDEIMBEMcBENEDOggIABCABBCxAzoICAAQsQMQgwE6CwguEIAEELEDENQCOg4ILhCABBCxAxDHARCjAjoFCC4QgAQ6BQgAEIAEOgUIABDLAToGCAAQHhAWOggIABAeEA8QFkoECEEYAEoECEYYAFCfBVjRGGCwImgDcAF4AIABdogB1gqSAQM1LjiYAQCgAQHIAQjAAQE&sclient=gws-wiz)

| 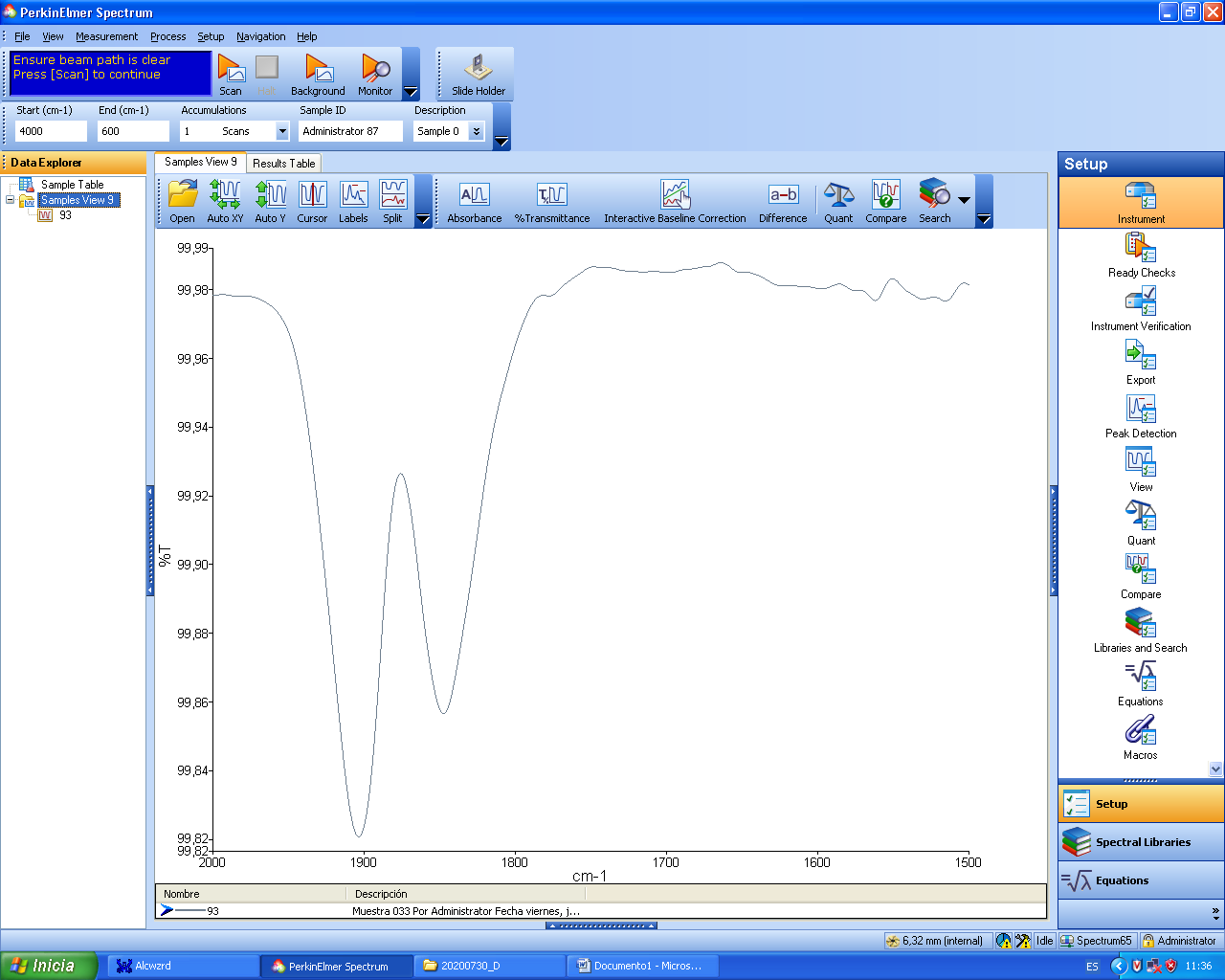**A** | 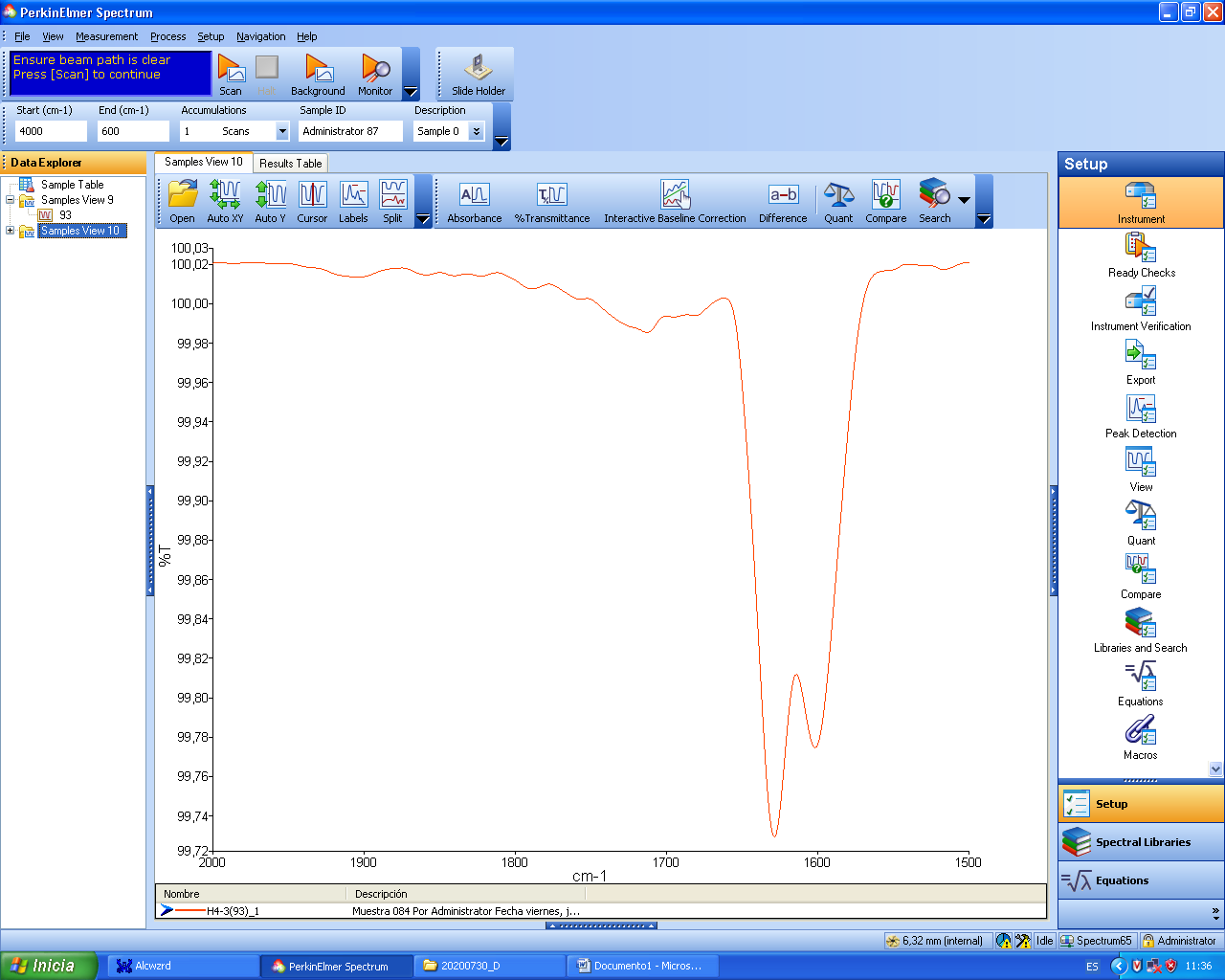**B** |
| --- | --- |
| 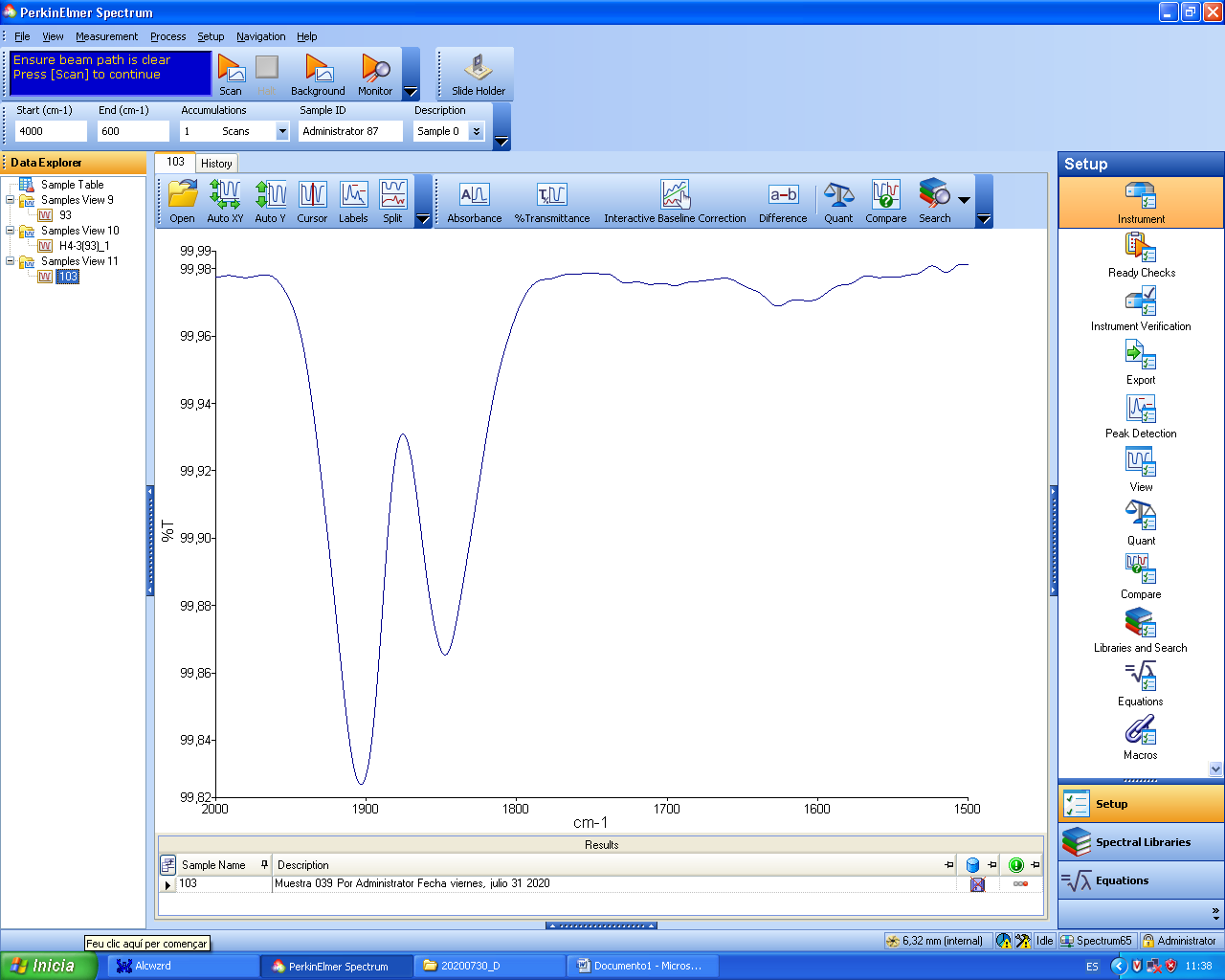**C** | 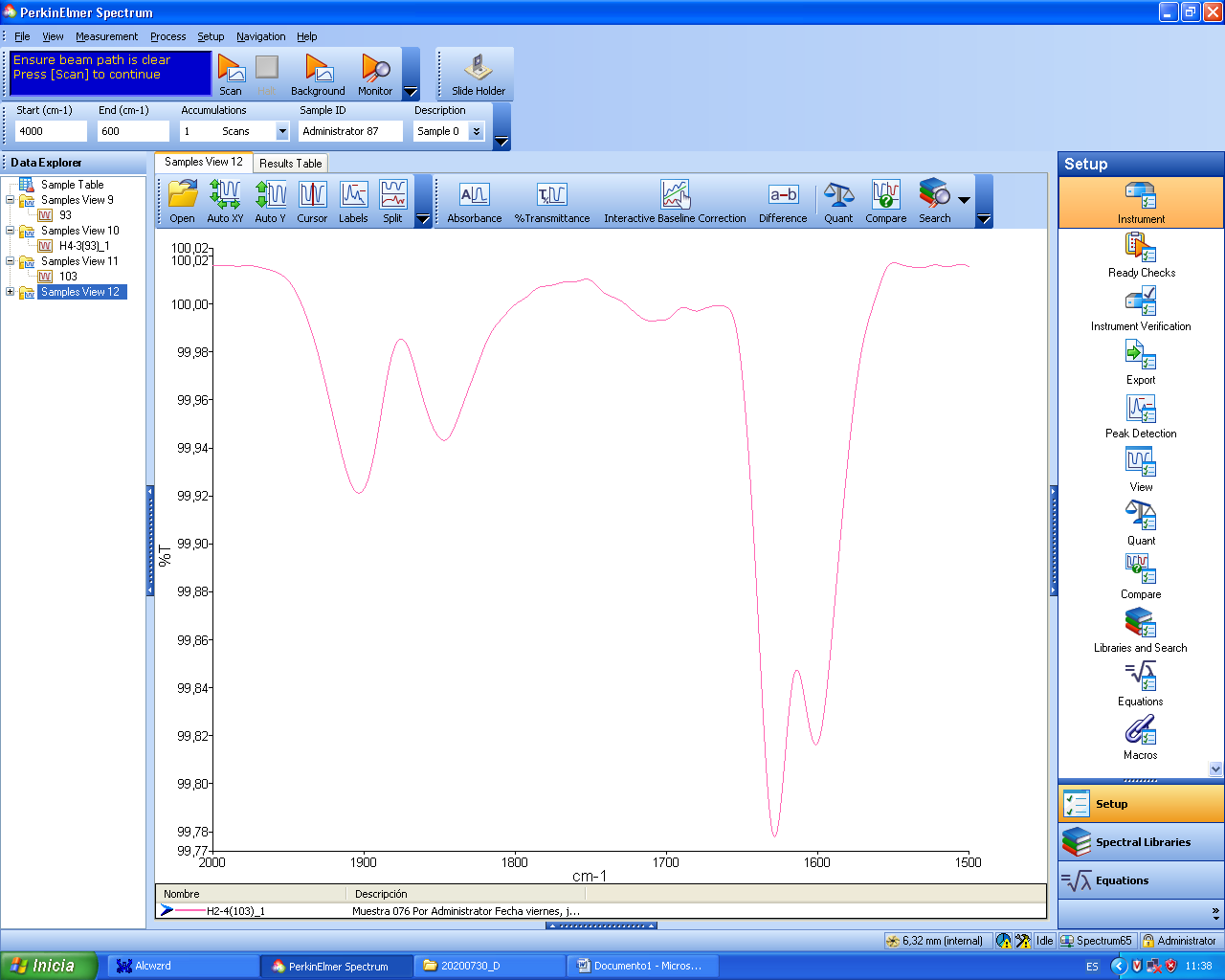**D** |

Figure S1 Fourier Transform Infrared Spectroscopy (FTIR) spectrum. HTX: (A) t= 0;(B) t = 1 h. HEX: (C) t=0 (D) t=1 h.

| 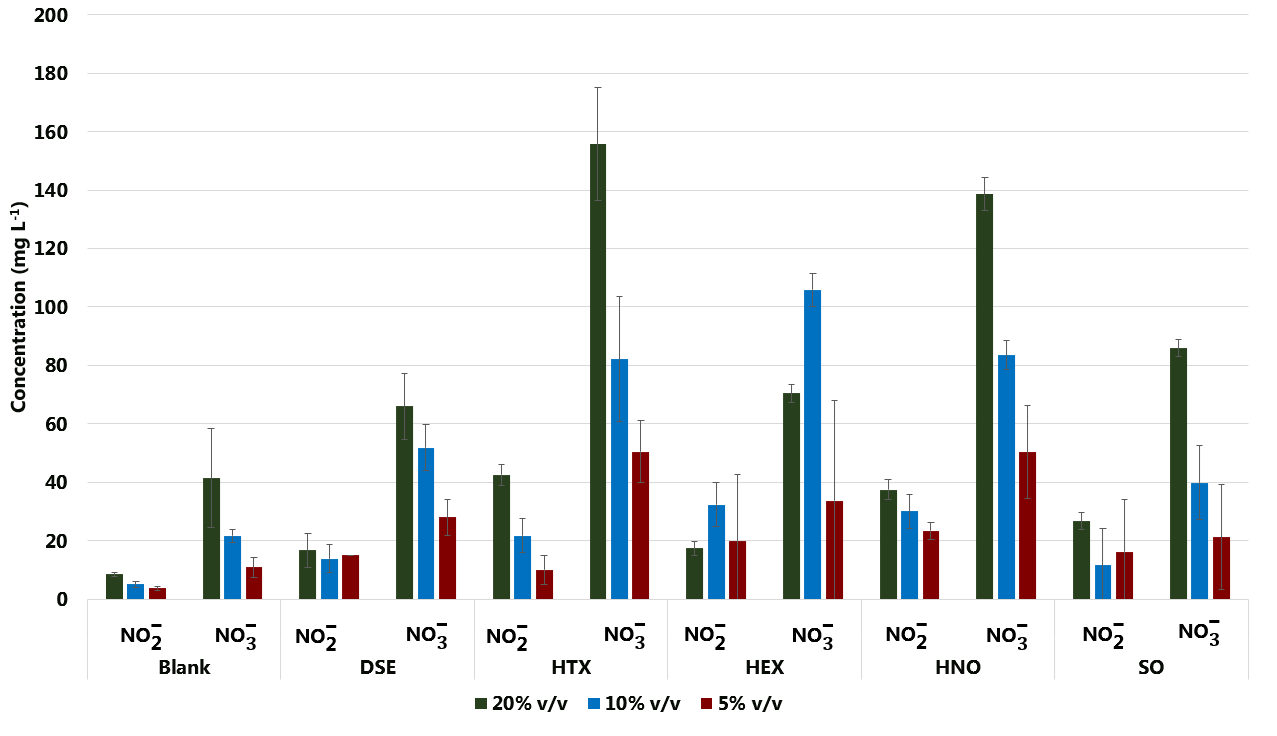**A** |
| --- |
| **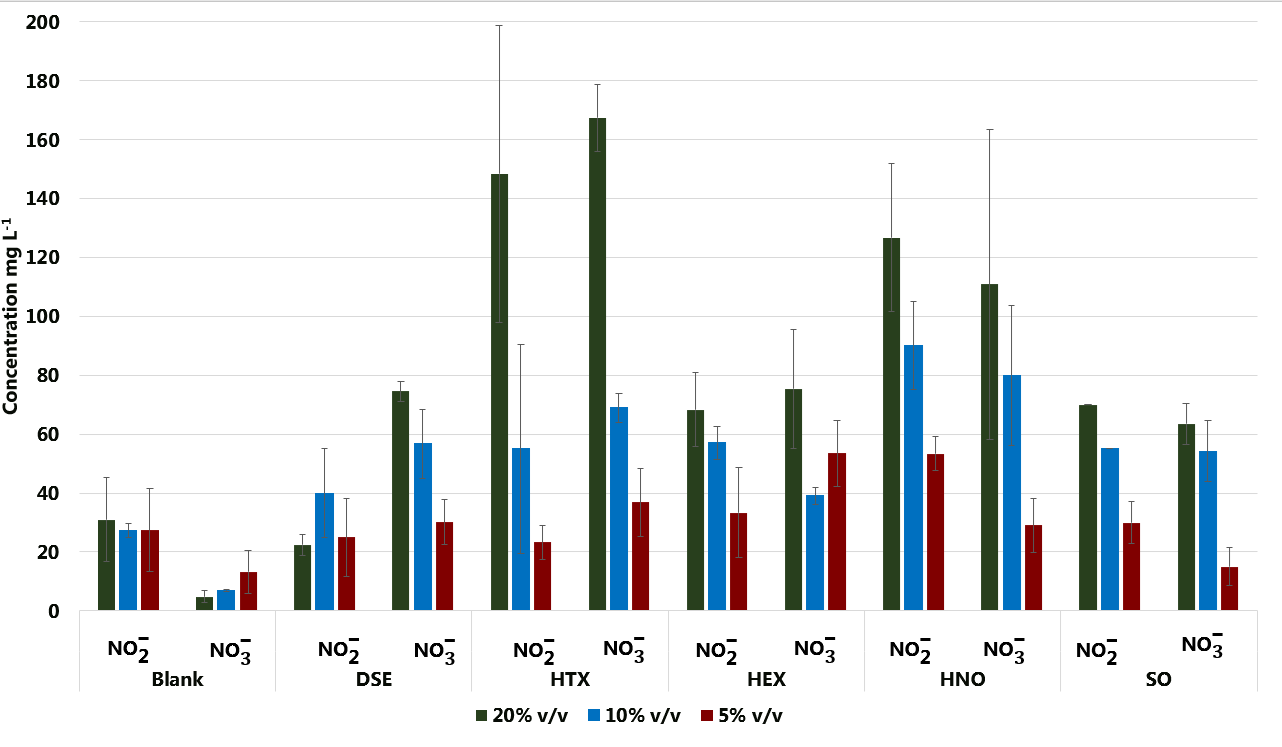B** |

Figure S2 Nitrite and nitrate concentration in a triphasic system. (A) water- (B) phosphate buffer.

| 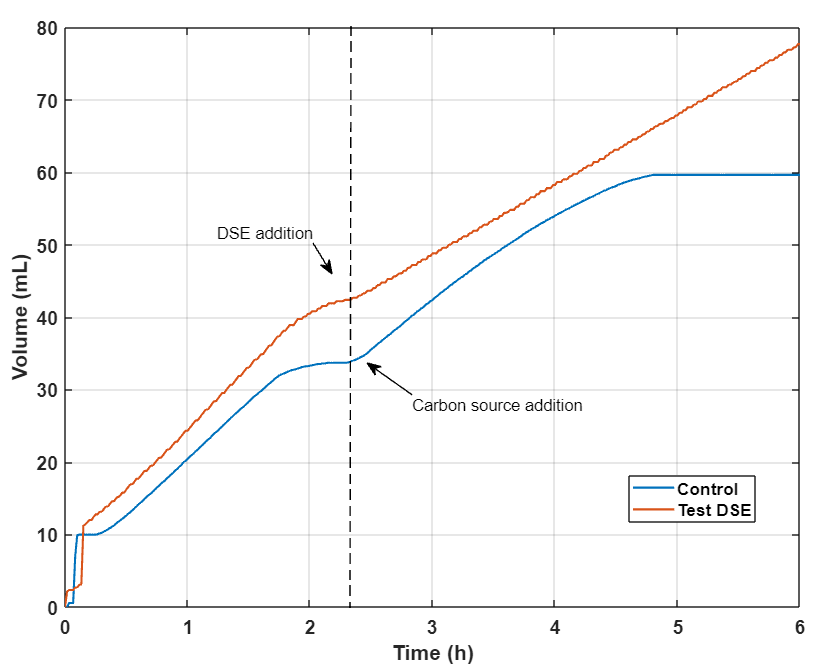**A** | 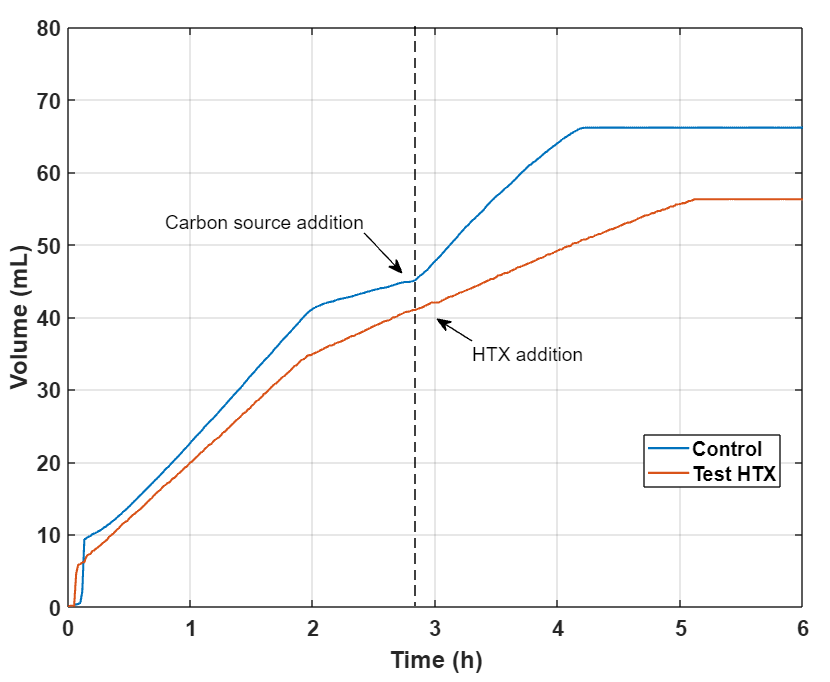**B** |
| --- | --- |
| 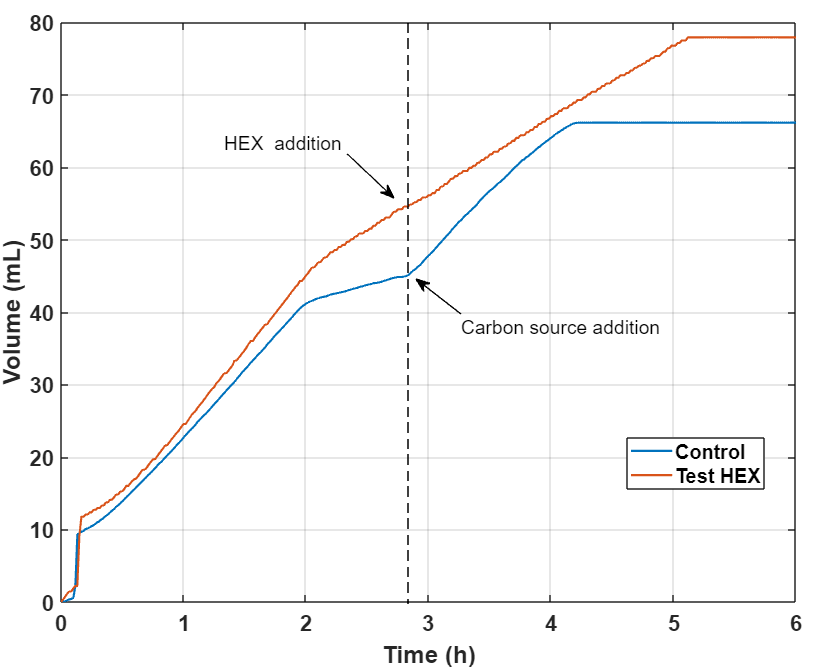**C** | **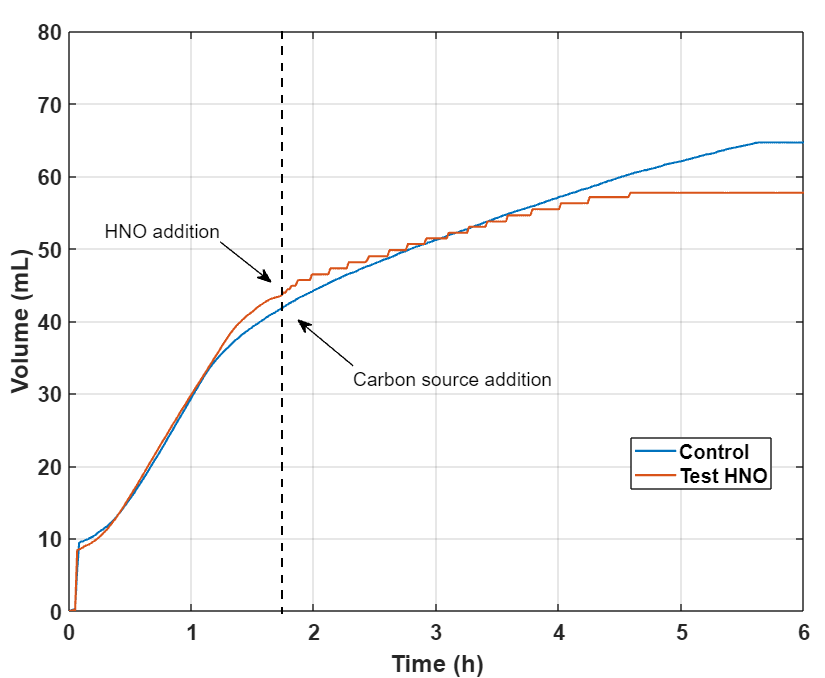D** |
| **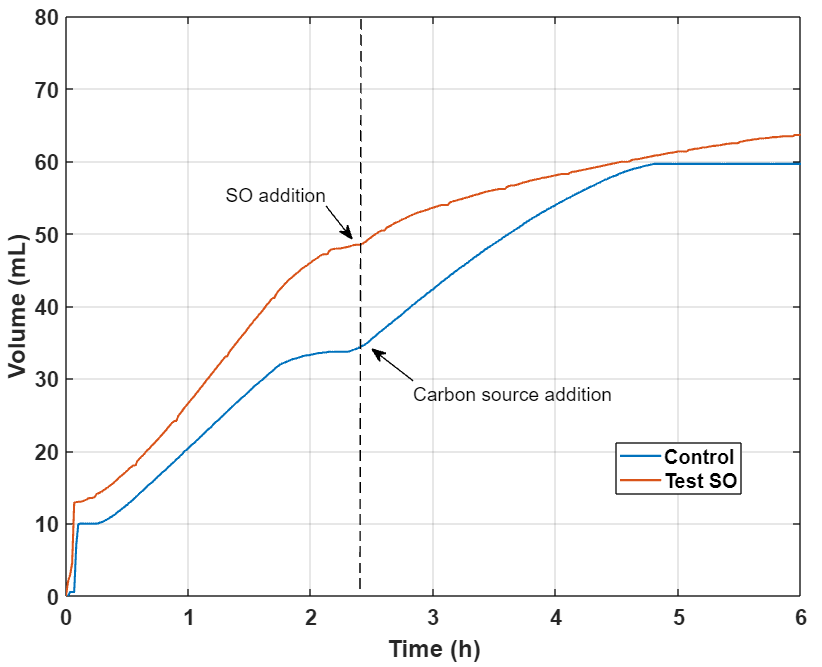E** |  |

Figure S3 Biodegradability test through denitrifying bacteria – Short term. (A) DSE, (B) HTX, (C) HEX, (D) HNO, (E) SO.
